# EEG and ECG Power Spectrum Analysis of Sedative Effects on Propofol-Anesthetized Rats with Electroacupuncture

**DOI:** 10.1155/2022/2440609

**Published:** 2022-05-27

**Authors:** Shu-ying Zhu, Jin Ma, Ze-ru Wang, Qing Chai, Liang-chun Yan, Jun Song, Jin-jun Shu, Huai-ming Wang, Yi-ding Chen

**Affiliations:** ^1^Department of Anesthesiology, Sichuan Cancer Hospital and Institute, Sichuan Cancer Center, School of Medicine, University of Electronic Science and Technology of China, Chengdu, Sichuan 610041, China; ^2^The Clinical Hospital of Chengdu Brain Science Institute, MOE Key Lab for Neuroinformation, Center for Information in Medicine, School of Life Science and Technology, University of Electronic Science and Technology of China, Chengdu, Sichuan 611731, China; ^3^Sichuan Academy of Chinese Medicine Sciences, Chengdu, Sichuan 610041, China

## Abstract

**Background:**

In previous studies, electroacupuncture (EA) with 2/15 Hz has been shown to enhance the sedative effects in general anesthesia patients. Central lateral thalamic stimulation of 50 Hz showed an arousal effect in macaques. Therefore, it is worth studying the sedative effect of EA at peripheral acupoints with different frequencies, especially the frequency of around 50 Hz.

**Methods:**

Rats were anesthetized under the constant infusion of propofol and EA at *Zusanli* (ST36) and *Neiguan* (PC6) locations. Electroencephalography (EEG) and heart rate were continuously recorded before and after the intervention by EA in the *C* group (control), *LEA* group (low-frequency group, 2/15 Hz diffuse/dense wave EA stimulation), and *HEA* group (high-frequency group, 50 Hz stimulation).

**Results:**

In the *LEA* group, a significant increase in the power of the delta component with a decrease in the alpha component (*p* < 0.05) was observed after EA stimulation. In the *HEA* group, significant increases in the powers of alpha and beta components of EEG (*p* < 0.05) and a decrease in the delta component of EEG were observed (*p* < 0.05). The phenomenon is also shown in full-frequency waves. In addition, a significant decrease in the low-frequency/high-frequency ratio parameter was observed in the *LEA* group.

**Conclusions:**

EA at bilateral ST36 and PC6 can enhance the sedative effects of propofol anesthesia in low-frequency stimulation but lighten the sedative effects in high-frequency (50 Hz) stimulation. The sympathetic-vagal balance was affected due to low-frequency EA.

## 1. Introduction

Various studies have shown that acupuncture could relieve preoperative anxiety and stress [1], lower blood pressure in mild hypertension patients [2], and improve diabetic gastroparesis [3]. However, these studies have only partially explored the sedation effects of acupuncture and its interaction with a sedative anesthetic.

Electroacupuncture (EA) is an effective technology that has demonstrated unique advantages in perioperative care. The depth of general anesthesia was shown to be significantly enhanced by EA at bilateral *Zusanli* (ST36) and *Neiguan* (PC6) locations in our previous study. [4] Hence, further studies are required to understand the underlying mechanism of the sedation effect of EA operated at different frequencies.

Electroencephalography (EEG) is a sensitive method used to detect changes in brain activity. Typical EEG waves vary when the brain is generating and losing consciousness. Studies on sleep have suggested that EEG changes from low-voltage fast activity to large delta (0.5–4 Hz) waves may cause a reduction in consciousness. [5] Propofol anesthesia appears to show a sleep-like state, sharing many features with slow-wave sleep. [6, 7] As for acupuncture, one study on healthy volunteers showed that theta-band power was significantly decreased during high-frequency acupuncture. [8] Another study on volunteers showed that delta energy was increased during acupuncture stimulation. [9] The above-mentioned observations show that EEG is effective in understanding the impact of EA on propofol sedation.

The diverse effects of acupuncture can be observed in the perioperative period. Acupuncture could reduce preoperative anxiety and stress [10] and achieve sedation. [11, 12] It can also shorten the waking-up time of the patient after surgery. [13] Diverse effects may be associated with different acupoints. Also, different frequencies of the electrical needle should be considered. A theory about anesthesia suggests a correlation of temporal binding and unbinding of 40 Hz oscillation with the perceptual task framework. [14] Cognitive unbinding appears if synchrony around 40 Hz is interrupted. [14, 15] Central lateral thalamic stimulation of 50 Hz can induce consciousness in macaques from stable anesthesia as per a recent study. [16] Therefore, EA around 50 Hz as a special stimulation at peripheral acupoints is worth studying.

Other physical signs, such as heart rate and heart rate variability (HRV), are also important to understand the effects of electroacupuncture. The ratio of the low-frequency component to the high-frequency component (LF/HF ratio) is one of the HRV indexes and can show EA effects on autonomic regulation. [17] In the present study, the following were investigated: (1) Changes in EEG in rats before and after electroacupuncture, based on propofol anesthesia, to further understand the role of EA sedation. (2) Whether low-frequency and high-frequency EA would have a different impact on EEG? What is the performance of a specific EA stimulation around 50 Hz acting on EEG? (3) The changes in heart rate and HRV in rats under different frequencies of EA stimulation.

## 2. Materials and Methods

### 2.1. Experimental Animals

A total of 36 Sprague–Dawley rats (200–250 g; SPF; Experimental Animal Center of Sichuan Academy of Chinese Medicine Sciences, China) were used in this study. These rats were raised at a room temperature of 20–22°C, relative humidity of 52%, and a 12-hour alternative light and dark cycle. A standard diet and water were provided.

A total of 2 ml/kg propofol (20 ml:0.2 g; Fresenius Kabi Deutschland GmbH) was injected in the tail vein to anesthetize the rats, and they were fixed to a stereotaxic holder. In addition, about 6.0 ml/kg∙h of propofol was continuously administered in the right femoral vein. This was performed by a venous constant-speed pump (Fresenius Kabi Deutschland GmbH).

### 2.2. EEG and ECG Recordings

Povidone-iodine solution and 75% ethanol were used to sterilize the skin after shaving the fur of the head. The skin was cut, the subcutaneous tissue and the periosteum were separated, and the anterior and posterior fontanelle were fully exposed. An electric drill (Strong 90, SAESHIN, Korea) was used to drill two small holes of 1.5–2.0 mm in diameter. One was located at the intersection of 1 mm in front of the coronal suture and 1 mm on the left side of the middle line of the skull. Another was located at the intersection of 1 mm in front of the lambdoid suture and 1 mm on the left side of the middle line. Two electrodes were screwed into the hole to make contact with the endocranium for EEG recordings.

Negative and positive electrodes were connected to the right forelimb and the left hindlimb for electrocardiogram (ECG) recordings. A physiological recorder (MP150, Biopic, USA) was used to record EEG and ECG.

### 2.3. EA Procedure

At the acupoints of bilateral *Zusanli* (ST36) and *Neiguan* (PC6), fine acupuncture needles (diameter: 0.35 mm, Hwato, China) were placed. On the basis of the rat spectrum, the location of acupoints was determined. ST36 is located at the anterolateral side of the hind limb, near the anterior tibial crest and under the anterior tibial muscle below the knee. PC6 is located at the proximal side of the accessory carpal pad of the forelimb between the flexor carpi radialis and the ligamentum palmaris longus [18]. The acupuncture needles were connected to the EA device (HANS-200A, Ji Sheng Medical, China).

All procedures complied with the National Institute of Health guidelines, and the Animal Care and Use Committee of Sichuan Cancer Hospital approved all the procedures performed in this study.

### 2.4. Experimental Protocol

**Figure 1:**
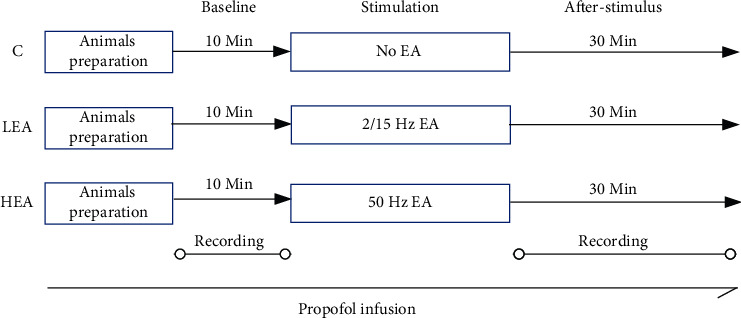
Experimental paradigm. After animal preparation, including EEG and ECG connections, EEG and ECG were recorded for 10 minutes, representing a signal baseline before stimulation. Then, the rats received different interventions according to groups. After stimulation, EEG and ECG were recorded for another 30 min. Anesthesia in rats was performed by constant propofol infusion.

Rats were divided into three groups, with an equal number of males and females in each group. For each group, EEG and ECG were recorded for 10 min prior to acupuncture interventions. The rats in group 1 were then given 30 min of low-frequency electroacupuncture on bilateral ST36 and PC6, which was a 2/15 Hz-2 mA diffuse/dense wave (*LEA group*). This wave density was the same as our previous study on patients. Rats in group 2 received 30 min of high-frequency electroacupuncture on bilateral ST36 and PC6, which specifically was a 50 Hz-2 mA wave (*HEA group*). Finally, the rats in group 3 received acupuncture without electric stimulation (*C group*). EEG and ECG recordings were suspended during the 30 min EA intervention in consideration of interference. After 30 min of stimulation, EEG and ECG were re-monitored for 30 min. The study protocol is schematically illustrated in Figure 1.

### 2.5. Data Analysis

#### 2.5.1. EEG Data Preprocessing

The time series in each data segment was removed during the EEG analysis before the following steps. In addition, each time series was normalized for further analysis.

#### 2.5.2. Power Spectrum Density (PSD) Analysis

A fast Fourier transform with a 500-sample Hanning window and 200-sample overlap was used to address this EEG issue. Then, the power of delta (1–4 Hz), theta (4–8 Hz), alpha (8–13 Hz), and beta (13–30 Hz) frequency bands was estimated separately. A proportion of each band for subsequent statistics was taken by the power of each band divided by the total power of the whole frequency band. These PSDs were calculated using Matlab (R2018b) scripts.

#### 2.5.3. ECG Processing

Acqknowledge 4.2 software was used to analyze the mean heart rate and HRV data. ECG recordings provided the HRV data. R waves were first identified, and then, R-R intervals were calculated. The R-R intervals were interpolated at 100 Hz and resampled at 8 Hz for spectral analysis. The low-frequency band was set as 0.04–1.0 Hz (LF), and the high-frequency band was set as 1.0–3.0 Hz (HF). Finally, the LF/HF ratio was calculated.

#### 2.5.4. Statistical Analysis

EEG statistical analysis was done using two different methods. First, the PSDs of each frequency point (1 Hz frequency resolution) before and after EA in each group were compared. Then, the power of four frequency components (delta, theta, alpha, and beta) before and after EA in each group was compared. The mean heart rate and the LF/HF ratio before and after EA were also compared in each group. All data were presented as mean ± SE. Student's *t*-test was used for comparison, and *P* < 0.05 was considered to be statistically significant.

## 3. Results

### 3.1. Effect of Low-Frequency EA on EEG Power in Propofol General Anesthesia

**Figure 2:**
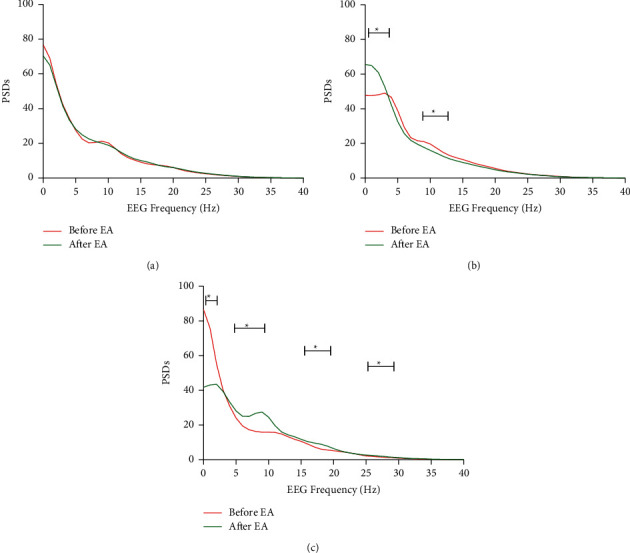
Full-frequency figure. PSDs of each frequency point (1 Hz frequency resolution). (a) Control group. (b) Low-frequency EA group. (c) High-frequency EA group. ^*∗*^*P* < 0.05, a significant difference between before and after EA. Short lines showed the frequency bands which had significant changes ((b) from left to right: 0–2 Hz and 9–12 Hz; (c) from left to right: 0–2 Hz, 6–10 Hz, 17–20 Hz, and 26–29 Hz).

**Figure 3:**
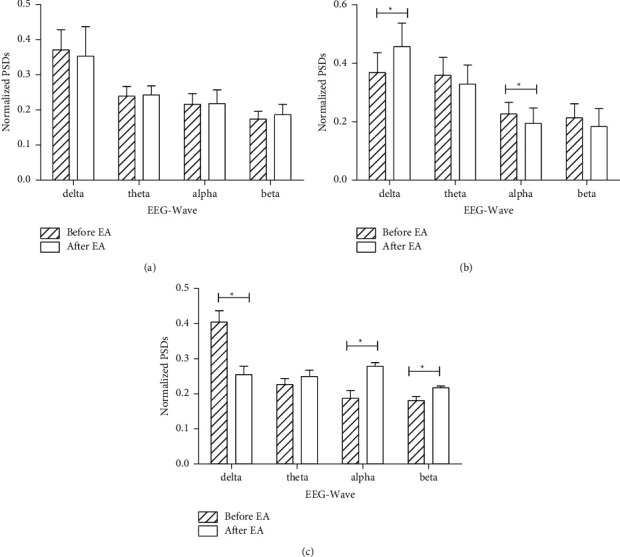
Normalized PSDs of delta, theta, alpha, and beta waves in three groups. The power of each band was divided by the total power of the whole frequency band to get a proportion of each band. (a) Control group. (b) Low-frequency EA group. (c) High-frequency EA group. ^*∗*^*P* < 0.05, a significant difference between before and after EA.

First, the PSDs of each frequency point (1 Hz frequency resolution) before and after EA were compared. No significant differences were observed at each frequency point (Figure 2(a)) in the *C* group. However, after 30 min of 2/15 Hz EA on bilateral ST36 and PC6, the power of low-frequency bands (0–2 Hz) increased and the power of high-frequency bands (9–12 Hz) decreased significantly (*p* < 0.05, Figure 2(b)). Furthermore, the power changes of the EEG on the classic bands (delta, theta, alpha, and beta) were analyzed. While comparing with the data before EA, the power of the delta component increased and the power of the alpha component decreased significantly (*p* < 0.05, Figure 3(b)).

### 3.2. Effect of High-Frequency EA on EEG Power in Propofol General Anesthesia

In the *HEA* group, a specific 50 Hz of EA was given to rats for 30 min. After stimulation, the power of low-frequency bands (0–2 Hz) decreased while the power of high-frequency bands (6–10 Hz, 17–20 Hz, 26–29 Hz) increased significantly in the full-frequency figure (*p* < 0.05, Figure 2(c)). Furthermore, the powers of alpha and beta components significantly increased after the stimulus (*p* < 0.05). In contrast, the delta component decreased significantly (*p* < 0.05, Figure 3(c)).

### 3.3. Effect of EA on Heart Rate and HRV under Propofol Anesthesia

**Figure 4:**
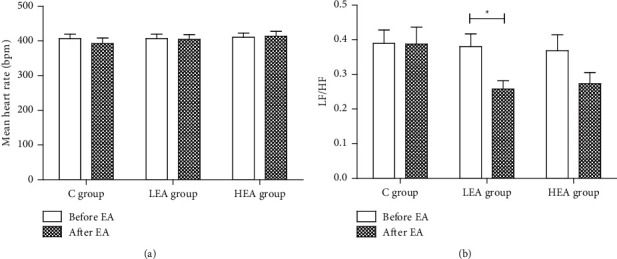
Effect of low-frequency and high-frequency EA on (a) mean heart rate and (b) LF/HF ratio in propofol anesthetic rats ^*∗*^*P* < 0.05, a significant difference between before and after EA.

The impact of low-frequency and high-frequency EA on the mean heart rate and HRV in propofol-anesthetized rats was investigated. Both the mean heart rate and LF/HF ratio showed no significant difference (*P* > 0.05, Figure 4) in the *C* group. However, after 30 min of EA stimulation with 2/15 Hz on bilateral ST36 and PC6 in the *LEA* group, the LF/HF ratio significantly decreased (*P* < 0.05, Figure 4(b)). This showed that the sympathetic component weakened while the vagal component increased in the autonomic activity after low-frequency EA stimuli. No significant changes in the mean heart rate were observed (Figure 4(a)). In the *HEA* group, the LF/HF ratio decreased without any significant changes (*P* > 0.05, Figure 4(b)). Also, the mean heart rate did not differ significantly compared to baseline (Figure 4(a)).

## 4. Discussion

In this study, EEG wave power, mean heart rate, and HRV before and after EA under propofol anesthesia were compared. Propofol is a widely used intravenous anesthesia in clinical practice. Propofol has a rapid onset and short half-time, making it an ideal anesthetic for intravenous pumping and providing stable blood concentration [19]. However, only a few articles based on the study about propofol anesthesia accompanied by EA exist. Previous studies on rats showed that a single bolus dose of propofol varied from 10 mg/kg to 40 mg/kg [20, 21]. In a study by Xing Y., continuous infusion of 50 mg/kg/h to 60 mg/kg/h of propofol could provide a suitable anesthetic state for rats [22]. In our study, a single bolus of 20 mg/kg and maintenance of 60 mg/kg/h were used in the pilot test. As seen in the control group, the heart rate of the rats was stable after one and a half hours with anesthesia. Other vital signs, such as breathing, were stable as well.

In this study, low-frequency stimulation of a 2/15 Hz diffuse/dense wave and high-frequency stimulation of 50 Hz were chosen. EA stimulation of a 2/15 Hz diffuse/dense wave has been proven to deepen the sedative effect in propofol-anesthetized patients. The acupuncture analgesia and sleeping mechanism involve endogenous opioid peptides. Studies have also shown that EA stimulation of both 2 Hz and 15 Hz increases the endorphin release in the brain, while EA stimulation of 15 Hz accelerates the release of other endogenous opioid peptides, such as endomorphin, dynorphin, and *β*-endorphin [23, 24]. On the other hand, cognitive unbinding of convergence is associated with anesthetic activity. The oscillatory activity near 40 Hz is related to primary sensory processing and reflects the temporal bind of the underlying cognition. [15] The interrupt coherence at around 40 Hz makes it unable for the neurons to synthesize the signals into a complete representation. [14] Central lateral thalamic stimulation of 50 Hz can induce consciousness in macaques from stable anesthesia as per a recent study. This was a frequency-specific effect since stimulation of 2 Hz, 10 Hz, and 200 Hz at the same site did not show this arousal effect[16]. 50 Hz stimulation at peripheral acupoints, ST36 and PC6, showed a consistent arousal effect on EEG in this study.

An increase in the delta-band components with a decrease in the alpha-band components was observed in the *LEA* group. However, in the *HEA* group, the delta-band components significantly decreased with a significant increase in the alpha and beta components. Significant differences in the full-frequency band figure were observed, which showed an increase in low-frequency bands (0–2 Hz) and a decrease in higher-frequency bands (9–12 Hz) in the *LEA* group and a decrease in low-frequency bands (0–2 Hz) and an increase in higher-frequency bands (6–10 Hz, 17–20 Hz, 26–29 Hz) in the *HEA* group. Delta waves are examined to access the depth of sleep under physiological conditions. The stronger the delta rhythm, the deeper the sleep. [25] Theta waves are associated with cognitive processing, such as forming and retrieving episodic and spatial memory. [26, 27] Alpha waves take over when an individual is in a state of relaxed wakefulness, and beta waves dominate the normal awake state, especially when an individual is attentive to decision-making or problem-solving. [28] Anesthesia shares many common characteristics with sleep. When propofol anesthesia is taken, the power of the delta range increases at a light anesthesia level. When propofol concentration was increased to a deep anesthesia level, the power of the alpha range lessened and delta or theta waves dominated. [9] Some studies indicated that interruption of the effective connection between the brain regions causes loss of consciousness, which eventually produces large slow waves. [29] Loss of consciousness is also due to a remarkable increase in slow waves. [29, 30] These changes reversed when consciousness was restored. [31] Thus, the increase of the delta component in the *LEA* group suggests that the EA stimulation of 2/15 Hz has a deepening sedative effect. This is consistent with our previous clinical trial results. However, the decrease of the delta component and increase of alpha and beta components in the *HEA* group suggest that electroacupuncture of 50 Hz lightened the anesthesia level.

In contrast, EA stimulation of 2/15 Hz and 50 Hz showed different effects on EEG in the anesthetized rats. This type of discrepancy was also observed in studies on EA and epilepsy-induced sleep disruption. For example, studies indicated that low-frequency EA at the *Feng Chi* acupoint improved epilepsy-induced sleep disruption, while high-frequency EA deteriorated the sleep disruption. [32] Furthermore, the study by Fei H showed that low-frequency EA stimulation (2 Hz) increased the release of met-enkephalin, but not dynorphin, while high-frequency EA stimulation (100 HZ) increased dynorphin rather than met-enkephalin. [33] These two are both endogenous opioid peptides. Therefore, the involvement of humoral factors may lead to different effects.

The noninvasive method to evaluate the balance of sympathetic and vagal activity used here is the heart rate variability. Among all the HRV indexes, the LF/HF ratio is considered a good indicator of the sympathetic-vagal balance. Here, LF and HF represent sympathetic activity and vagal modulation. [34] Electroacupuncture has significantly reduced the LF/HF ratio in the *LEA* group in this study. No change in the mean heart rate was observed. In the *HEA* group, the degree of LF/HF ratio reduction appeared to be weakened (*p*=0.054). No significant difference in the mean heart rate was observed. However, this result was partly consistent with some previous clinical studies. Increased vagal activity and a reduced LF/HF ratio in both healthy and postoperative patients were observed in acupuncture at the auricular point. [35, 36] A significant decrease in the LF/HF ratio after stimulation of ST36 was shown in another study on anesthetic rats. [18] It is speculated that the somatosympathetic reflex may be involved in the EA regulation mechanism. The somatosympathetic reflex occurs between the body surface and the visceral organs of the same nerve segment. [37] Through this reflex, acupuncture at an acupoint may have autonomic regulation on the visceral organs of the same nerve segment. This study showed that 2/15 Hz EA had an autonomic regulation on propofol-anesthetized rats, and it was frequency-dependent.

This study has certain limitations. This study did not include a stimulation group higher than that of 50 Hz. Therefore, the reason for the reduced sedative effects, whether dependent on 50 Hz or not, is still not clear. Further studies are needed to confirm this fact. Furthermore, different techniques, including multichannel EEG, histology, and functional magnetic resonance imaging, can be used in future studies. To extend the generalizability, further studies should be performed on human subjects. We assume that these steps will help in further understanding the sedative and arousal effects of EA.

## 5. Conclusion

This study suggested that EA at bilateral *Zusanli* (ST36) and *Neiguan* (PC6) acupoints can enhance the sedative effects on propofol anesthesia in low-frequency stimulation but reduce the sedative effects in high-frequency stimulation (50 Hz). Low-frequency EA affected the sympathetic-vagal balance, which increased vagal activity and decreased sympathetic activity in ratios. In light of these results, the effectiveness of the sedative effects of EA in the perioperative period is promising.

## Data Availability

The datasets generated and analyzed during the present study are available from the corresponding author upon reasonable request.

## Abbreviations

EA: Electroacupuncture
ST36: *Zusanli* acupoint
PC6: *Neiguan* acupoint
EEG: Electroencephalography
ECG: Electrocardiogram
HRV: Heart rate viability
LF/HF: Low-frequency component to the high-frequency component.
